# Optical coherence tomography-based machine learning for predicting fractional flow reserve in intermediate coronary stenosis: a feasibility study

**DOI:** 10.1038/s41598-020-77507-y

**Published:** 2020-11-24

**Authors:** Jung-Joon Cha, Tran Dinh Son, Jinyong Ha, Jung-Sun Kim, Sung-Jin Hong, Chul-Min Ahn, Byeong-Keuk Kim, Young-Guk Ko, Donghoon Choi, Myeong-Ki Hong, Yangsoo Jang

**Affiliations:** 1 Division of Cardiology, Cardiovascular Center, Korea University Anam Hospital, Korea University College of Medicine, Seoul, Korea; 2 Department of Electrical Engineering, Sejong University, Neungdong-ro 209, Gwangjin-gu, Seoul, 05007 Korea; 3 Division of Cardiology, Severance Cardiovascular Hospital, Yonsei University Health System, Yonsei University College of Medicine, Yonsei-ro 50-1, Seodaemun-gu, Seoul, 03722 Korea; 4 Cardiovascular Research Institute, Yonsei University College of Medicine, Seoul, Korea; 5 Severance Biomedical Science Institute, Yonsei University College of Medicine, Seoul, Korea

**Keywords:** Interventional cardiology, Cardiovascular diseases

## Abstract

Machine learning approaches using intravascular optical coherence tomography (OCT) to predict fractional flow reserve (FFR) have not been investigated. Both OCT and FFR data were obtained for left anterior descending artery lesions in 125 patients. Training and testing groups were partitioned in the ratio of 5:1. The OCT-based machine learning-FFR was derived for the testing group and compared with wire-based FFR in terms of ischemia diagnosis (FFR ≤ 0.8). The OCT-based machine learning-FFR showed good correlation (r = 0.853, *P* < 0.001) with the wire-based FFR. The sensitivity, specificity, positive predictive value, negative predictive value, and accuracy of the OCT-based machine learning-FFR for the testing group were 100%, 92.9%, 87.5%, 100%, and 95.2%, respectively. The OCT-based machine learning-FFR can be used to simultaneously acquire information on both image and functional modalities using one procedure, suggesting that it may provide optimized treatments for intermediate coronary artery stenosis.

## Introduction

Fractional flow reserve (FFR) is a functional assessment with high specificity and used to diagnose myocardial ischemia in an unreliable angiographic luminal narrowing. However, when considering percutaneous coronary intervention (PCI) for ischemia based on FFR, the lack of anatomical information on atherosclerotic plaques can be problematic in patients, especially those with acute coronary syndrome^1^. Meanwhile, intravascular optical coherence tomography (OCT), which is a high-resolution imaging modality, can provide the morphological information about lesion characteristics more accurately than angiography and intravascular ultrasound. OCT and FFR are applied differently for coronary interventions, such as to guide decision-making during coronary revascularization (FFR) and procedure optimization (OCT). In context, the combination of OCT and FFR measurements may provide additional information to guide the application of an appropriate treatment strategy. However, using both strategies in all clinical practices increases time and cost. Therefore, using the combination of OCT and FFR measurements with imaging-based physiological parameters is beneficial. Previous studies reported that the simulations of OCT-derived computational flow dynamics (CFD) allowed additional functional estimates of FFR, demonstrating a good correlation with invasive FFR measurements^2–5^. However, the CFD of FFR derived from coronary imaging may have limited applications in clinical practice because of limited OCT coronary geometry-based CFD modeling such as absence of geometry of side branches and prolonged simulation time of 3D coronary geometry reconstruction and CFD^2^. Recently, machine learning models for the prediction of FFR based on angiography^6^ and intravascular ultrasound^7^ have been reported. However, the use of a machine learning approach based on OCT studies has not yet been investigated. This study aims to evaluate and compare the diagnostic accuracy of the machine learning-FFR based on OCT with wire-based FFR.

## Results

### Clinical and lesion characteristics

The mean age of the subjects was 63 years. About 75% of the study population was male, and diabetes mellitus was diagnosed in 30% of the subjects. No statistical significance was observed in the comparison of clinical characteristics between the training and the testing groups (Supplementary Table S1). Similarly, no statistical significance was observed between the two groups in terms of the OCT characteristics (Supplementary Table S2).

### Major features of the OCT-based machine learning-FFR

**Figure 1:**
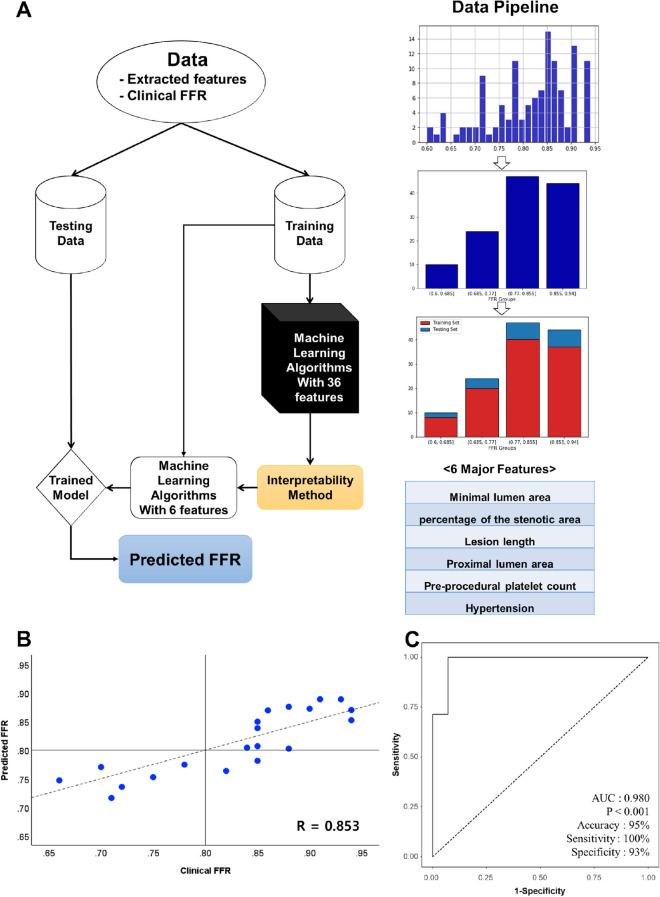
Optical coherence tomography-based machine learning for predicting fractional flow reserve. (**A**) Flow chart of the proposed machine learning method. (**B**) Comparison between the clinical fractional flow reserve results and the predicted fractional flow reserve results by the Random Forest model in the testing set. (**C**) Receiver operating characteristic curve of machine learning-fractional flow reserve. *FFR* fractional flow reserve, *AUC* area under the curve.

A total of 36 features were defined. They are summarized in Table 1. In the testing samples, the Random Forest model, using the six most important features (based on weight), namely, minimal LA, percentage of the stenotic area, lesion length, proximal LA, pre-procedural platelet count, and hypertension, obtained the highest performance (*r* = 0.853) (Fig. 1A).

**Table 1:** List of 36 features, their weight, and standard deviation.

	Feature	Weight	Standard deviation
1	Minimal lumen area	0.431489	0.201828
2	Area stenosis (%)	0.115880	0.038884
3	Lesion length	0.035337	0.011430
4	Pre-procedural platelet count	0.033187	0.021882
5	Proximal lumen area	0.026289	0.004752
6	Hypertension	0.016973	0.006676
7	Distal lumen area	0.009928	0.015942
8	Pre-procedural blood urea nitrogen level	0.007642	0.007495
9	Hypercholesterolemia	0.002688	0.002036
10	Calcified nodule	0.002309	0.000532
11	Pre-procedural hemoglobin level	0.001440	0.010278
12	Fibrocalcific nodule	0.000846	0.001332
13	Lipid rich plaque	0.000843	0.000886
14	Existence of thrombus	0.000077	0.001775
15	Dissection	0.000008	0.000292
16	lipid arc over 90° with thickness less than 65 μm	0.000000	0.000000
17	Existence of ruptured plaque	− 0.000032	0.002259
18	Diabetes mellitus	− 0.000096	0.001015
19	Age	− 0.000137	0.004589
20	Existence of erosion	− 0.000268	0.000213
21	Weight	− 0.000353	0.007105
22	lipid arc over 90°	− 0.000460	0.002299
23	Existence of macrophage	− 0.000802	0.004656
24	Unstable angina	− 0.000820	0.003374
25	Fibrous nodule	− 0.000922	0.001797
26	Existence of necrotic core	− 0.000950	0.000307
27	Gender	− 0.001616	0.000551
28	Existence of cholesterol crystal	− 0.002124	0.001706
29	Current smoking	− 0.003752	0.002504
30	Pre-procedural creatinine level	− 0.004177	0.012168
31	Existence of microvessels	− 0.004760	0.001435
32	Body mass index	− 0.006832	0.002180
33	Systolic blood pressure	− 0.008183	0.004773
34	diastolic blood pressure	− 0.008704	0.000831
35	Plaque area	− 0.011278	0.017001
36	Height	− 0.024011	0.013424

### Performance of the OCT-based machine learning-FFR

Figure 1B illustrates the predicted results of the Random Forest model using the six most important features compared to the clinical FFR of the testing set. The results showed a good correlation (*r* = 0.853, *P* < 0.001) and agreement (MAE = 0.04) between the OCT-based machine learning-FFR and the wire-based FFR. In the analysis of the Bland–Altman plot, the statistical limits of the OCT-based machine learning-FFR was 0.01 ± 0.11, based on the wire-based FFR (Supplemental Fig. S1). Based on an FFR ≤ 0.8, the sensitivity, specificity, positive predictive value, negative predictive value, and accuracy of the OCT-based machine learning-FFR method for the testing group were 100%, 92.9%, 87.5%, 100%, and 95.2%, respectively (Fig. 1C). Also, the positive and negative likelihood ratios were 14 and 0, respectively.

## Discussion

In clinical practice, machine learning-based approaches have been used to complement existing prediction models by analyzing associations between numerous variables. In this study, we developed a machine learning-FFR method to predict the functional ischemia of a stenotic coronary artery using patient information and OCT images. To our knowledge, this is the first OCT-based machine learning-FFR assessment.

In current clinical practice, FFR and OCT are widely used for coronary intervention regarding decision-making of coronary revascularization (FFR) and procedure optimization (OCT), respectively. The simultaneous use of both modalities during PCI might be expected to achieve better clinical outcomes. However, there are limitations to conducting both tests because of time, cost, and lack of evidences^8^. Although OCT-guided PCI demonstrated stent optimization and greater procedural success compared to IVUS and angiographic-guided PCI in ILUMIEN III trial, there were no differences in 30-day and one-year clinical outcomes because of relatively small number of patients^9,10^. Additionally, more than two-thirds of operators were found to have based PCI decisions on angiographic findings after considering prolonged procedure times, cost, and risk of complications^11^.

To overcome discrepancies between the guidelines and the actual clinical practice, methods to measure FFR such as computational tomography imaging (CT-FFR) or OCT imaging (OCT-FFR) have been proposed by using CFD^2,12^. When comparing OCT-FFR to OCT-based machine learning-FFR for the patients in this study, the OCT-based machine learning-FFR (r = 0.853) had better correlation compared with CFD-based OCT FFR (r = 0.712) on the same set of patients. In addition, even considering different patient population and vessel characteristics, the OCT-based machine learning-FFR demonstrated a better or comparative result to the wire-based FFR results than did OCT-FFR (r = 0.83)^13^ or CT-FFR (r = 0.82)^12^. These findings suggested that the OCT-based machine learning-FFR results could be used to predict FFR as an alternative method to both CT-FFR and OCT-FFR.

Recently, machine learning had been introduced in FFR measurements for cardiovascular imaging. Machine learning angiography and machine learning-based intravascular ultrasound (IVUS) results had good correlations with the wire-based FFR results and acceptable diagnostic accuracy^6,7^. Coenen et al. reported that ML-based CT-FFR closely reproduces CFD-based CT-FFR calculations. Although CFD-based CT-FFR has a good correlation with FFR, the processing times of CFD algorithms vary with their complexity, which remains a limitation. However, the ML-based CT-FFR calculations can be performed virtually without delay. Moreover, the diagnostic performance of ML-based CT-FFR can improve with better image quality^14^. In this study, OCT features were used as a feature of machine learning. The actual contour of the lumen, as viewed on a high-resolution image, is one of the most important factors in CFD simulations^2^. Thus, we suggested that the superiority of OCT, in terms of resolution, to CT, angiography, and IVUS image^15^ had an advantage in the diagnostic performance of machine learning-FFR. In addition, the OCT based machine learning-FFR could provide an accurate analysis of both the lesion characteristics and functional significance of the lesion.

The question of what modality to use for intermediate coronary lesions is still unanswered due to different advantages and disadvantages of image techniques and functional assessments. Although there are no randomized clinical data, OCT-guided interventions have been introduced as promising tools for patients with stable coronary artery disease as well as for those with acute coronary syndrome^16^. A recent study reported that OCT-based PCI had a lower rate of both major adverse cardiac events and significant angina than those of FFR-based PCI, suggesting the importance of the assessments of the coronary vessel anatomy^17^. However, the cost-effectiveness of FFR and its power to determine the status of ischemia are still important in daily practice. In context, various machine learning-based FFR were developed based on the image modalities^6,7^. But, in previous machine learning FFR studies, the impact of clinical characteristics on machine learning FFR has been underestimated. Thus, we suggested that patient clinical characteristics that have underestimated might affect the evaluation of FFR. Further investigation is needed to clarify this issue. In this study, the OCT-based machine learning-FFR method was used to perform a functional assessment of the coronary artery based on patient information and OCT data, resulting in a good correlation with wire-based FFR. This finding suggested that the use of the OCT-based machine learning-FFR method could simultaneously acquire information on both image and functional modalities using one invasive procedure, and in turn, might provide optimized treatments for intermediate coronary artery stenosis as well as save time and cost.

This study had several limitations. It was a small cohort study at a single center only for patients with intermediate lesions in the left descending artery and thus more clinical data will be required to expand this method to other coronary arteries to remove potential bias of the present results. Also, because of the small number of subjects, it seems that there is a pattern towards higher FFR than OCT-based ML-FFR when FFR value increases in the Bland–Altman plot. However, despite its small number, this was the first study on the OCT-based machine learning-FFR method. In addition, since OCT acquires a high-resolution image of the actual contour of the lumen, which is one of the most critical factors in CFD simulations compared to angiography or CT image, OCT-based machine learning FFR may have in prospect better results compared to other modalities-based machine learning FFR. Thus, a large study should be conducted to assess method performance and accuracy. Moreover, this study analyzed only patients who had lesions in their left anterior descending artery. Because of the relatively small number, we excluded different territories that might act as a confounder. In the IVUS-based ML model and CFD study, which were analyzed for multiple lesion locations, the diagnostic performance was relatively low compared to that in this study, in which LAD selection was performed^7,18^. Although this location resulted in a better correlation between the anatomical and the functional parameters compared to other locations^19^, further study is needed to expand this model to other coronary locations. Also, side branch information variables were not included in this study. The impact of the size and location of side branches should be investigated. In addition, some OCT features among 6 major features were obtained manually (percentage of the stenotic area and lesion length. However, intra-observer variability analysis and inter-observer variability analysis revealed acceptable reliability for measurement (Supplemental Table S3). In terms of measurement time, OCT-derived FFR computation took about 20 min due to the manual procedure of OCT lumen extraction and 3D rendering for CFD. However, OCT-based machine learning FFR took 2–3 min to extract key OCT features and analyze FFR. It is unclear whether this machine learning method, principally based on some selected area measurements, will be superior to the current practice of determining the degree of stenosis visually or quantitatively. However, the Pearson correlation between percentage of the stenotic area and the wire-based FFR was inferior compared to that of the OCT-based machine learning-FFR (*r* = 0.469 vs. *r* = 0.853). Besides, the Pearson correlation between minimal LA in OCT and the wire-based FFR was relatively inferior compared to that of OCT-based machine learning-FFR (*r* = 0.545 vs. *r* = 0.853). Thus, despite the limitations, we suggest that OCT-based machine learning-FFR may provide optimized treatments for intermediate lesions in the left descending artery.

## Methods

A total of 141 consecutive patients who had undergone both OCT and FFR during their evaluations of intermediate stenosis in the left anterior descending artery, between November 2013 and January 2015, were enrolled in the Yonsei OCT registry (ClinicalTrials.gov, NCT02099162). Sixteen patients were excluded because of poor OCT images (A suboptimal OCT image quality because of insufficient blood clearance (n = 13) and improper coverage of the entire lesion by OCT (n = 3)). A total of 125 patients were finally included in the analysis. The inclusion criteria were: (1) typical angina, (2) a de novo lesion of intermediate stenosis (diameter stenosis = 40–70%) in the left anterior descending artery from the proximal to the middle portions, and (3) a lesion length less than 20 mm as shown by angiography. The exclusion criteria were: (1) hypersensitivity to the contrast agent, (2) use of inotropic agents due to hemodynamic instability, (3) severe ventricular dysfunction (left ventricular ejection rate < 30%), (4) creatinine level greater than or equal to 2.0 mg/dL, (5) life expectancy less than 12 months due to noncardiac comorbidity, and (6) severe heart valve disease. This study was approved by the institutional review board at Severance Hospital and complied with the Declaration of Helsinki. Written informed consent was obtained from all patients.

### OCT measurements

OCT images were obtained using a frequency-domain OCT system (C7-XR OCT imaging system, LightLab Imaging, Inc./Abbott Vascular, Chicago, IL, USA). OCT cross-sectional images were acquired at a rate of 100 frames/s. The fiber probe was retracted at a velocity of 20 mm/s from the stationary imaging sheath. Analysts who were blinded to the patient and procedural information in the core laboratory (Cardiovascular Research Center) analyzed the OCT data. The minimal luminal area (LA) was defined as the segment with the smallest LA by OCT analysis. The proximal reference LA and the distal reference LA were the region within the same segment as the lesion with the largest lumen. Both reference LA were usually within 10 mm of the stenosis without major intervening branches^20^. The minimal LA used to define functional stenosis for the OCT criteria was 1.96 mm^2,21^. The percentage of the stenotic area (%) was defined as [(mean reference LA − minimum LA)/mean reference LA] × 100. In this study, the OCT analysis of the lesions and a detailed explanation of analyzed features were based on previous OCT studies^22,23^.

### Wire-based FFR measurements

Using a 0.014-inch pressure guidewire (Abbott Vascular, Chicago, IL, USA), coronary artery pressure was measured during coronary angiography. The pressure guidewire was positioned distal to the target lesion after performing equalization. To induce maximal hyperemia, 140 μg/kg/min intravenous adenosine was administered via the antecubital vein. FFR was calculated using the following formula: mean hyperemic distal coronary pressure/mean aortic pressure. Functionally significant stenosis was defined as an FFR ≤ 0.8. A pressure drift of ± 3 mm Hg was considered acceptable. If the pressure drift exceeded this margin, the FFR recording was repeated.

### Feature selection

A total of 36 features were used to develop the OCT-based machine learning-FFR approach in this study. The features for developing a machine learning FFR model of coronary intermediate lesion were selected according to the expert opinion by worldwide guidelines and prior literature search. In the guidelines of the American Society of Cardiology and the European Society of Cardiology, patients’ age, sex, heart rate, blood pressure (BP), and past medical history were used in clinical decision making for ischemic heart disease^24,25^. In addition, OCT features were selected from prior literature search that investigated the correlation between coronary artery disease and OCT characteristics^22,23^. The extracted features included OCT geometric and biometric features. For data normalization, we used the standard score to scale the value of extracted features to reduce the effect of outlier data points^26^. These features, used as inputs for the machine learning model to estimate FFR, are illustrated in Table 1: two epidemiological data points (gender, age), five clinical data points at the time of admission to the cardiac catheterization laboratory (systolic blood pressure, diastolic blood pressure, height, weight, and body mass index), nine past medical history items (history of unstable angina, hypertension, diabetes mellitus, dyslipidemia, smoking, and four laboratory test results prior to presenting with chest pain—platelet count, levels of hemoglobin, blood urea nitrogen, and creatinine), and 20 OCT data points (proximal LA, minimal LA, distal LA, lesion length, plaque area, percentage of the stenotic area, presence of atheroma (fibrous, fibro-calcific, and lipid rich), lipid arc greater than 90°, lipid arc greater than 90° with a thickness less than 65 µm, presence of dissection, presence of a necrotic core, presence of microvessels, presence of cholesterol crystals, presence of rupture, presence of erosion, presence of calcified nodules, presence of macrophages, and presence of thrombi).

### Machine learning-FFR assessment based on OCT

The supervised machine learning framework performed according to the following steps: (1) feature extraction, (2) applying the machine learning algorithm, and (3) assessing permutation feature importance. In this study, Random Forest^27^ was used to estimate FFR.

In general, training and testing samples were required for constructing and evaluating the supervised machine learning model. Before assigning the data to the training and testing groups, a stratified sampling technique^28^ was utilized to divide the data into four distinctive subgroups to prevent the chance of omitting one sub-group and thus leading to sampling bias^29^. The four sub-groups of FFR values were: (0.600, 0.685) with 10 subjects, (0.685, 0.770) with 24 subjects, (0.770, 0.855) with 47 subjects, and (0.855, 0.940) with 44 subjects. Data were assigned to the training and testing sets in the ratio of 5:1 (Fig. 1A). In the machine learning training phase, 104 patients were trained offline using the machine learning-based algorithm and 36 extracted features (clinical features, lesion characteristics, and OCT features). In the case of Random forest optimization, a technique of cross validation (CV) was performed on the training set to optimize for hyperparameter tuning. The training set was split into K number of subsets, called folds, and a fitting Random forest with K = 4 was applied. A Random forest approach was performed using many iterations of the entire four-fold CV process, each time using different hyperparameter combination settings. The four optimal hyperparameters values of Random forest algorithms are summarized in Table 2 (n_estimators = 1000, max_depth = 50, min_samples_leaf = 2 and min_samples_split = 2) and default values of the other remaining parameters were utilized. Once the optimized parameter values were chosen, a model was constructed using the chosen parameters, and then evaluated using the testing set.

**Table 2:** Random Forest parameters.

Optimized hyperparameters	Description	Value
N_estimators	Number of trees in Random forest	1000
Max_depth	Maximum number of levels in tree	50
Min_samples_split	Minimum number of samples required to split a node	2
Min_samples_leaf	Minimum number of samples required at each leaf node	2

The best Random Forest model with optimal hyperparameters was then selected. The feature importance from RF model were calculated based on the training data given to the model. Here, permutation importance, introduced by Breiman^27^, was used to measure the increase in the error of the prediction model after permuting the feature’s values. To achieve the best in testing, we choose top 6 feature of final RF model for training set. In the testing phase, 21 patients with 6 important features were tested online using the trained models to predict FFR. During the evaluation of our experiments, the Pearson correlation coefficient and the mean absolute error (MAE) were used to evaluate the Random forest model. The MAE between the clinical FFR and the predicted FFR is defined below:

$$ MAE = \frac{1}{n}\sum\limits_{i = 1}^{n} {\left| {\hat{y}_{i} - y_{i} } \right|} , $$

where *n* denotes the number of cases; $y_{i}$, the clinical FFR; and $\hat{y}_{i}$, the predicted FFR.

## Supplementary information

Supplementary Information

## Data Availability

The datasets analyzed during the current study are available from the corresponding author on reasonable request.
