# Supplementary material for: Optical coherence tomography-based machine learning for predicting fractional flow reserve in intermediate coronary stenosis: a feasibility study
Source: Sci Rep. 2020 Nov 24;10:20421. doi: 10.1038/s41598-020-77507-y (PMC7686372; doi:10.1038/s41598-020-77507-y)
Supplement: Supplementary file 1 — Supplementary Information [file 41598_2020_77507_MOESM1_ESM.docx]

Optical Coherence Tomography-based Machine Learning for Predicting Fractional Flow Reserve in Intermediate Coronary Stenosis: A Feasibility Study

Jung-Joon Cha^1^, Tran Dinh Son^2^, Jinyong Ha^2,†^, Jung-Sun Kim^3,4,*^, Sung-Jin Hong^3^, Chul-Min Ahn^3,4^, Byeong-Keuk Kim^3,4^, Young-Guk Ko^3,4^, Donghoon Choi^3,4^, Myeong-Ki Hong^3,4,5^, and Yangsoo Jang^3,4,5^

^1^Division of Cardiology, Cardiovascular Center, Korea University Anam Hospital, Korea University College of Medicine, Seoul, Korea

^2^Department of Electrical Engineering, Sejong University, Seoul, Korea

^3^Severance Cardiovascular Hospital, Yonsei University Health System, Seoul, Korea

^4^Cardiovascular Research Institute, Yonsei University College of Medicine, Seoul, Korea

^5^Severance Biomedical Science Institute, Yonsei University College of Medicine, Seoul, Korea

Jung-Joon Cha, Tran Dinh Son, Jinyong Ha, and Jung-Sun Kim contributed equally to this manuscript. Correspondence and requests for materials should be addressed to J.H.(jinyongha@sejong.ac.kr) or J.-S.K.(kjs1218@yuhs.ac)

Address Co-correspondence to:

Jung-Sun Kim, MD, PhD

*Division of Cardiology, Severance Cardiovascular Hospital, Yonsei University College of Medicine, 03722 Yonsei-ro 50-1, Seodaemun-gu, Seoul, Korea

Telephone: (82-2)-2228-8457, Fax: (82-2)-393-2041,

E-mail: kjs1218@yuhs.ac

Jinyong Ha, PhD

†Department of Electrical Engineering, Sejong University, 05007 Neungdong-ro 209, Gwangjin-gu, Seoul, Korea

Telephone: (82-2)-3408-3959, Fax: (82-2)-3408-4337,

E-mail: jinyongha@sejong.ac.kr

Supplemental Materials

Supplementary Figure 1. The Bland-Altman plot between clinical FFR and OCT-based machine learning FFR.


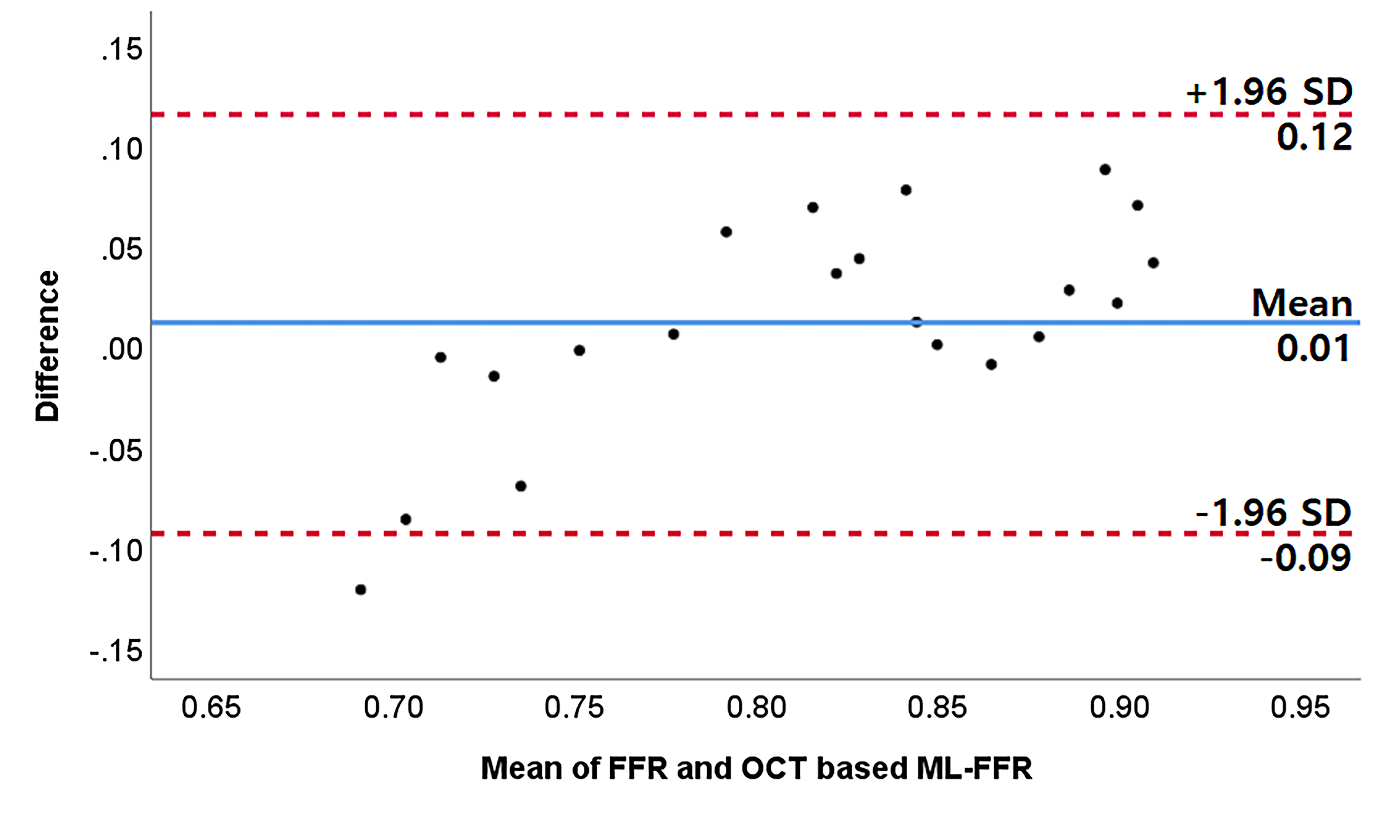


Supplemental Table 1 Comparison of clinical characteristics between the training and testing groups.

|  | Total | Training | Testing | p |
| --- | --- | --- | --- | --- |
| Age (years) | 62.7 ± 9.1 | 62.5 ± 9.3 | 63.6 ± 7.8 | 0.595 |
| Male, n (%) | 94 (75.2) | 79 (76.0) | 15 (71.4) | 0.782 |
| Unstable angina, n (%) | 40 (32.0) | 34 (32.7) | 6 (28.6) | 0.802 |
| Diabetes mellitus, n (%) | 39 (31.2) | 30 (28.8) | 9 (42.9) | 0.301 |
| Hypertension, n (%) | 77 (61.6) | 66 (63.5) | 11 (52.4) | 0.461 |
| Hypercholesterolemia, n (%) | 59 (47.2) | 48 (46.2) | 11 (52.4) | 0.639 |
| Current smoking, n (%) | 28 (22.4) | 24 (23.1) | 4 (19.0) | 0.782 |
| Body mass index (kg/m^2^) | 25.0 ± 3.1 | 25.1 ± 3.1 | 24.6 ± 3.0 | 0.508 |
| Height (cm) | 165.6 ± 8.1 | 165.6 ± 8.0 | 165.6 ± 8.8 | 0.986 |
| Weight (kg) | 68.7 ± 10.8 | 68.9 ± 11.0 | 67.6 ± 10.2 | 0.625 |
| Systolic blood pressure (mmHg) | 129.9 ± 21.9 | 130.5 ± 22.3 | 126.8 ± 19.5 | 0.473 |
| Diastolic blood pressure (mmHg) | 71.3 ± 11.0 | 71.2 ± 11.4 | 71.4 ± 8.5 | 0.921 |
| Pre-procedural platelet count ($\boldsymbol{\times}$10^3^μL) | 237.2 ± 60.3 | 237.6 ± 62.7 | 235.4 ± 46.7 | 0.885 |
| Pre-procedural hemoglobin level (mg/dL) | 14.2 ± 1.7 | 14.2 ± 1.8 | 14.6 ± 1.2 | 0.382 |
| Pre-procedural BUN level (mg/dL) | 15.5 ± 3.7 | 15.6 ± 3.9 | 15.1 ± 2.6 | 0.452 |
| Pre-procedural creatinine level (mg/dL) | 0.83 ± 0.15 | 0.84 ± 0.16 | 0.79 ± 0.15 | 0.286 |

Data are expressed as mean (SD) or number (percentage), where appropriate. Continuous variables were compared using an independent t-test and categorical variables were compared using the Chi-square test.

Supplemental Table 2 Comparison of OCT characteristics between the training and testing groups.

|  | Total | Training | Testing | p |
| --- | --- | --- | --- | --- |
| Proximal lumen area (mm) | 7.6 ± 2.9 | 7.4 ± 2.7 | 8.7 ± 3.6 | 0.067 |
| Minimal lumen area (mm) | 2.4 ± 1.2 | 2.3 ± 1.2 | 2.5 ± 1.5 | 0.577 |
| Distal lumen area (mm) | 5.9 ± 2.5 | 5.8 ± 2.6 | 6.0 ± 2.1 | 0.839 |
| Lesion length (mm) | 23.2 ± 8.5 | 23.5 ± 8.3 | 21.6 ± 9.6 | 0.343 |
| Plaque area | 15.1 ± 4.7 | 14.9 ± 5.0 | 15.8 ± 3.1 | 0.406 |
| Area stenosis (%) | 83.8 ± 7.4 | 83.7 ± 7.0 | 84.1 ± 9.2 | 0.829 |
| Fibrous nodule, n (%) | 24 (19.2) | 19 (18.3) | 5 (23.8) | 0.374 |
| Fibrocalcific nodule, n (%) | 48 (38.4) | 41 (39.4) | 7 (33.3) | 0.634 |
| Calcified nodule, n (%) | 13 (10.4) | 11 (10.6) | 2 (9.5) | 1.000 |
| Lipid rich plaque, n (%) | 53 (42.4) | 44 (42.3) | 9 (42.9) | 1.000 |
| Lipid arc over 90 degree, n (%) | 32 (25.6) | 25 (24.0) | 7 (33.3) | 0.414 |
| lipid arc over 90 degree with thickness less than 65 um, n (%) | 3 (2.4) | 3 (2.9) | 0 (0.0) | 0.573 |
| Existence of dissection, n (%) | 4 (3.2) | 3 (2.9) | 1 (4.8) | 0.526 |
| Existence of necrotic core, n (%) | 14 (11.2) | 12(11.5) | 2 (9.5) | 0.571 |
| Existence of microvessels, n (%) | 52 (41.6) | 44 (42.3) | 8 (38.1) | 0.811 |
| Existence of cholesterol crystal, n (%) | 46 (36.8) | 38 (36.5) | 8 (38.1) | 1.000 |
| Existence of Rupture, n (%) | 11 (8.8) | 9 (8.7) | 2 (9.5) | 1.000 |
| Existence of Erosion, n (%) | 12 (9.6) | 11 (10.6) | 1 (4.8) | 0.689 |
| Existence of macrophage, n (%) | 53 (42.4) | 45 (43.3) | 8 (38.1) | 0.810 |
| Existence of thrombus, n (%) | 19 (15.2) | 17 (16.3) | 2 (9.5) | 0.739 |

Data are expressed as mean (SD) or number (percentage), where appropriate. Continuous variables were compared using an independent t-test and categorical variables were compared using the Chi-square test.

Supplemental table 3. Intra-observer variability analysis and inter-observer variability analysis of OCT features

| Correlation coefficient | Intra-observer  variability | Inter-observer  variability |
| --- | --- | --- |
| Minimal lumen area | 0.987 | 0.993 |
| Percentage of the stenotic area | 0.980 | 0.977 |
| Lesion length | 0.949 | 0.946 |
| Proximal lumen area | 0.993 | 0.994 |

OCT; optical coherence tomography
